# Evaluating the Efficacy of Tension Band Wiring Fixation for Chaput Tubercle Fractures

**DOI:** 10.3390/jcm12175490

**Published:** 2023-08-24

**Authors:** Sung-Joon Yoon, Eui-Dong Yeo, Ki-Jin Jung, Yong-Cheol Hong, Chang-Hwa Hong, Sung-Hun Won, Kyung-Jin Lee, Jae-Young Ji, Je-Yeon Byeon, Dhong-Won Lee, Woo-Jong Kim

**Affiliations:** 1 Department of Orthopaedic Surgery, Soonchunhyang University Hospital Cheonan, 31, Suncheonhyang 6-gil, Dongam-gu, Cheonan 31151, Republic of Korea; yunsj0103@naver.com (S.-J.Y.); c89546@schmc.ac.kr (K.-J.J.); ryanhong90@gmail.com (Y.-C.H.); chhong@schmc.ac.kr (C.-H.H.); 2 Department of Orthopaedic Surgery, Veterans Health Service Medical Center, Seoul 05368, Republic of Korea; angel_doctor@naver.com; 3 Department of Orthopaedic Surgery, Soonchunhyang University Hospital Seoul, 59, Daesagwan-ro, Yongsan-gu, Seoul 04401, Republic of Korea; orthowon@schmc.ac.kr; 4 Department of Orthopaedic Surgery, Soonchunhyang University Hospital Bucheon, 170, Jomaru-ro, Bucheon-si 14584, Republic of Korea; leekj6840@naver.com; 5 Department of Anesthesiology and Pain Medicine, Soonchunhyang University Hospital Cheonan, 31, Suncheonhyang 6-gil, Dongam-gu, Cheonan 31151, Republic of Korea; phmjjy@naver.com; 6 Department of Plastic Surgery, Soonchunhyang University Hospital Cheonan, 31, Suncheonhyang 6-gil, Dongam-gu, Cheonan 31151, Republic of Korea; wpdusqus@gmail.com; 7 Department of Orthopaedic Surgery, Konkuk University Medical Center, 120-1, Neungdong-ro, Gwangjin-gu, Seoul 05030, Republic of Korea; bestal@naver.com

**Keywords:** ankle fracture, syndesmosis injury, chaput tubercle, avulsion fracture, tension band wiring

## Abstract

Background: Chaput tubercle fractures, located at the attachment site of the anterior inferior tibiofibular ligament (AITFL) on the distal tibia, have the potential to destabilize the syndesmosis joint. This study aims to assess the effectiveness of tension band wiring (TBW) as a surgical intervention for managing Chaput fractures and the consequent syndesmosis instability. Methods: A retrospective review of patient charts was undertaken for those who had undergone ankle fracture surgery from April 2019 through May 2022. The surgical procedure involved direct fixation of the Chaput fractures using the TBW method. Radiological assessments were performed using postoperative simple radiographs and computed tomography (CT) scans, while clinical outcomes were evaluated using the Olerud–Molander Ankle Score (OMAS) and the visual analog scale (VAS). Results: The study included 21 patients. The average OMAS improved significantly, rising from 5.95 preoperatively to 83.57 postoperatively. Similarly, the average VAS score dropped from 7.95 before the surgery to 0.19 thereafter. Minor wound complications were reported by three patients, and one case of superficial infection was resolved with antibiotic therapy. Conclusions: Our findings suggest that the TBW technique is an effective surgical approach for treating Chaput fractures and associated syndesmosis instability. It provides reliable fixation strength and leads to improved long-term functional outcomes. Further research is needed to compare the TBW technique with alternative methods and optimize the treatment strategies for these complex ankle fractures.

## 1. Introduction

Ankle fractures frequently involve damage to surrounding soft tissue and ligaments, which can significantly impair ankle joint stability and functionality [1]. Therefore, the comprehensive treatment of ligament damage, alongside anatomical reduction and the internal fixation of bone fractures, is paramount for optimal clinical and radiological outcomes [2,3]. Within this context, distal tibiofibular syndesmosis is crucial for maintaining ankle congruency and integrity under weight-bearing conditions, warranting surgical intervention in cases of instability [4]. Of the various ligaments within the ankle joint, the anterior inferior tibiofibular ligament (AITFL) is particularly vital for maintaining stability [5,6]. The Chaput tubercle, which serves as the AITFL attachment site, is susceptible to fractures—commonly termed “Chaput fractures”—leading to syndesmosis joint instability [7]. Recent studies have suggested that direct fixation represents the optimal treatment for syndesmosis joint instability induced by Chaput fractures [8,9,10]. Although K-wires and screws are commonly employed for direct fixation, tension band wiring (TBW) serves as an advantageous alternative given its superior fixation strength [10,11]. In a previous study, Yeo et al. [12] suggested a novel tension band wiring technique for Chaput fractures, which has proven to be a reliable method for treating Chaput fractures and associated syndesmosis instability, thereby improving long-term functional outcomes. This paper presents the results of surgeries employing this technique in the treatment of Chaput fractures.

## 2. Materials and Methods

### 2.1. Patient Selection

The Institutional Review Board of Soonchunhyang University Hospital, Cheonan, Republic of Korea, approved this study (IRB No. 2023–07–018). A retrospective chart review was conducted on patients who underwent ankle fracture surgery from April 2019 to May 2023. Fractures were diagnosed using anteroposterior (AP), lateral, and mortise X-rays of both ankles, in addition to 3D computed tomography (CT) scans. The Lauge–Hansen classification method was used to categorize ankle fracture patterns, while the modified Wagstaffe classification method classified patterns of AITFL avulsion fractures. Only patients with a follow-up period exceeding 1 year were included in the study. We excluded patients under 18 years old, those with an ipsilateral fracture extending to the tibial plafond, and those with a history of contralateral ankle fracture or syndesmotic injury, as well as cases involving open fractures.

### 2.2. Clinical Evaluations

Postoperative follow-ups were conducted at 2 weeks, 4 weeks, 6 weeks, 3 months, 6 months, and 12 months. The Olerud–Molander Ankle Score (OMAS) was calculated for all patients to assess overall functionality and subjective satisfaction following ankle injuries [13]. Pain was measured using the visual analog scale (VAS). The scoring of the patients was evaluated preoperatively and at the last follow-up ≥12 months postoperatively.

### 2.3. Radiological Evaluations

**Figure 1:**
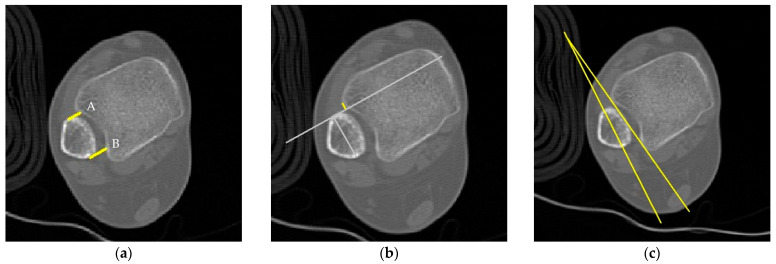
Radiologic measurements: (**a**) direct anterior difference (A) and direct posterior difference (B); (**b**) fibular translation; (**c**) fibular rotation.

Syndesmosis reduction was radiologically evaluated using simple radiographs and postoperative axial CT images taken 1 cm proximal to the tibial plafond. Four radiographic measurements were selected for assessment, with a picture archiving and communication system (PACS; Dejaview, Dongeun Information Technology, Bucheon, Republic of Korea) employed to obtain the necessary measurements (Table 1 and Figure 1) [14,15,16,17,18,19,20,21]. To ensure objectivity, two independent observers evaluated each patient’s four radiographic measurements. After a 6-week interval, these measurements were repeated. The observers were blinded to the patients’ clinical outcomes and provided with initial treatment details, excluding current patient complaints.

### 2.4. Surgery

**Figure 2:**
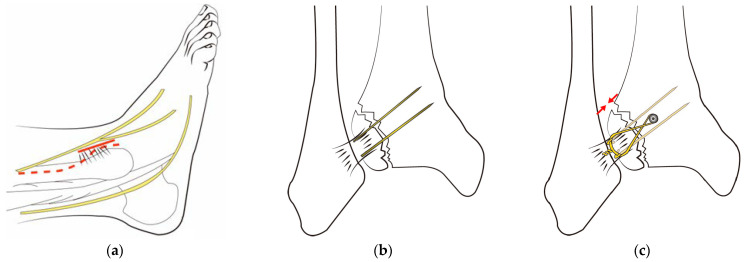
Surgical technique for Chaput tubercle fracture: (**a**) For a fibular fracture, a curved anterolateral approach (indicated by the dotted line) is used to expose the fracture site. In the absence of a fibular fracture, a small anterolateral incision (approximately 2–3 cm) is made directly over the palpable Chaput tubercle on the tibia’s lower end (shown as a solid line). (**b**) After reducing the Chaput fracture, fixation is achieved with two K-wires and screws. (**c**) Stainless-steel wires are looped around both the screw and the K-wires in a figure-of-eight fashion. Reproduced from ED Yeo et al., Medicina (Kaunas), 2022 Jul 27;58(8):1005, with permission from WJ Kim [12].

**Figure 3:**
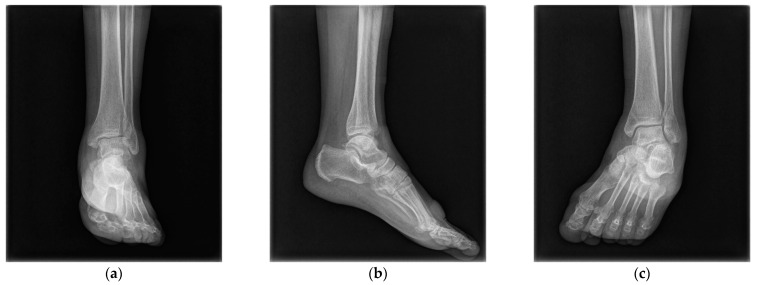
Preoperative anteroposterior (**a**), lateral (**b**), and mortise (**c**) X-ray images of a 47-year-old woman’s left ankle, revealing a short oblique fracture of the lateral malleolus and a Chaput fracture.

**Figure 4:**
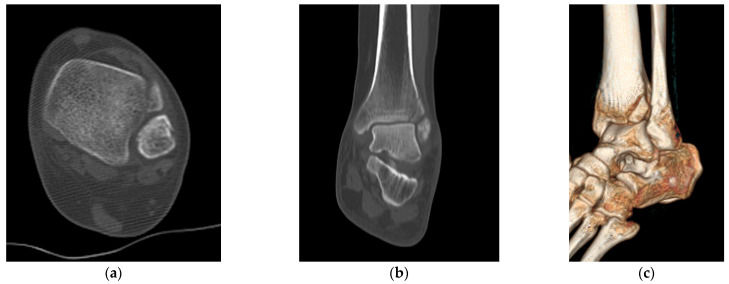
Preoperative axial (**a**), coronal (**b**), and three-dimensional (**c**) computed tomography images illustrating the Chaput fracture.

**Figure 5:**
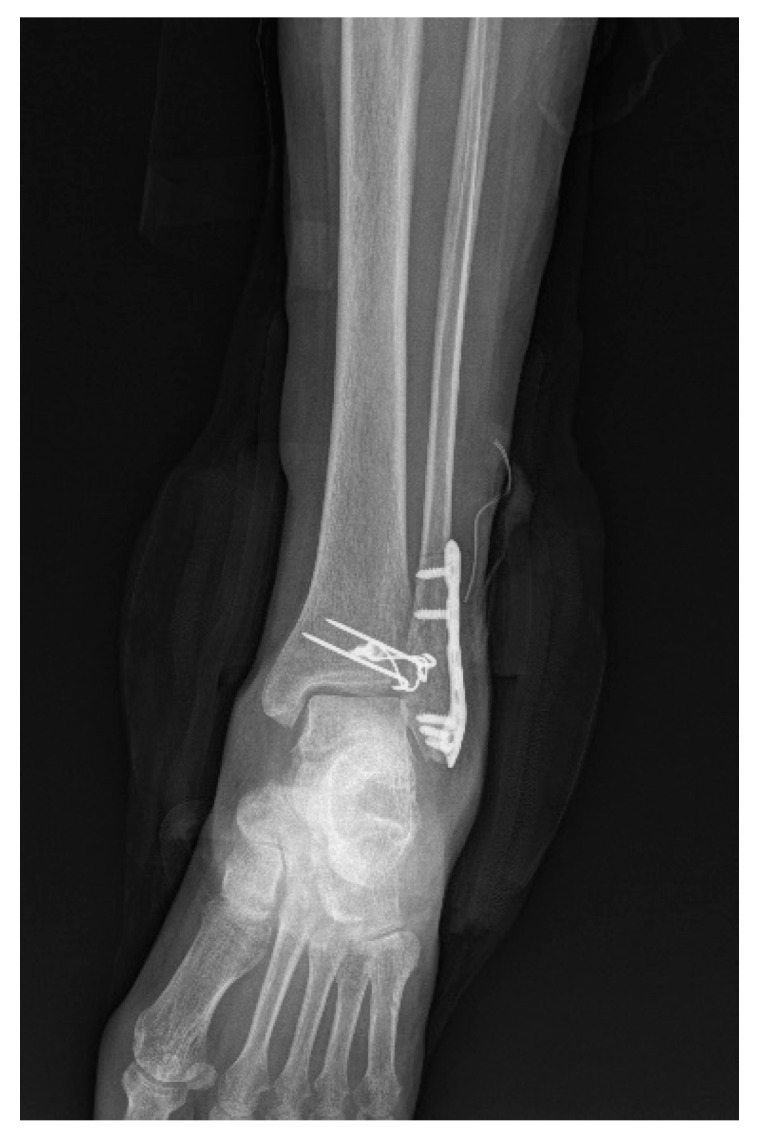
Postoperative X-ray of a Chaput fracture treated using the tension band wiring technique.

**Figure 6:**
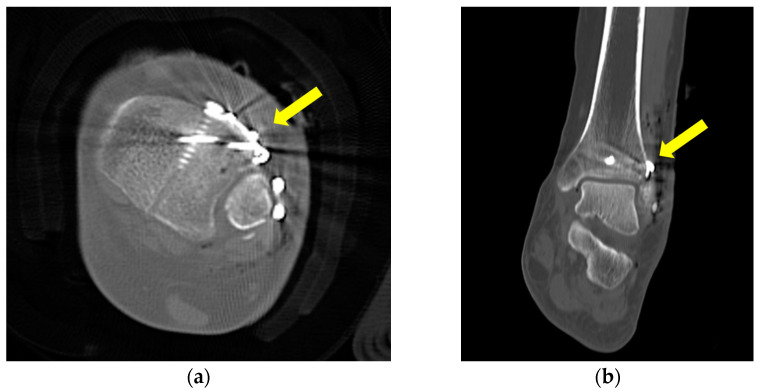
Postoperative axial (**a**) and coronal (**b**) computed tomography images demonstrating successful reduction, compression, and fixation of the Chaput fracture, as indicated by the arrow.

The same surgeon performed all procedures. The patient was positioned supine under general or spinal anesthesia or a lower extremity nerve block. The surgical site was sterilized, and a tourniquet was inflated for clear surgical field visualization. For fibular fractures, a curved anterolateral approach was employed for plate reduction and fixation. For non-fibular fractures, a small anterolateral incision was made over the palpable Chaput tubercle of the distal tibia. The Chaput fracture fragment was cleared of debris and reduced using small point-reduction forceps. Intraoperative fluoroscopy confirmed appropriate hardware positioning, congruency of articular surfaces, and no displacement of these surfaces. To anchor a figure-of-eight wire distally and prevent fracture rotation, two K-wires (1.2–1.6 mm in diameter) were inserted proximally through the fracture site from the end edge of the Chaput fragment. After bending the ends, the K-wires were slightly pulled back to ensure full seating on the tubercle end. A medial incision over the distal tibia was retracted to reveal the anterolateral tibial border approximately 2–3 cm above the fracture site. A cancellous full-thread screw (4.0 mm in diameter) was inserted untapped and without complete seating, while stainless-steel wires (0.8 mm in diameter) were looped around both the screw and K-wires in a figure-of-eight fashion. The loops were tightened to cling to the anteroinferior surfaces of the distal Chaput fragments, and the steel wires were twisted at their K-wire insertion points. Finally, two K-wires were obliquely cut, bent medially, and tapped into medial malleoli, with thinner K-wires and steel wires used for smaller fracture fragments (Figure 2). Figure 3 and Figure 4 display preoperative plain X-ray and CT images of a 47-year-old woman with a Chaput fracture. Figure 5 shows a postoperative plain X-ray image of a Chaput fracture treated with open reduction and internal fixation using the outlined technique. Axial CT images further confirmed the reduction and fixation, as shown in Figure 6.

### 2.5. Statistical Analysis

A statistical expert performed the statistical analysis. All calculations were carried out using the SPSS software (version 26.0; IBM Corp., Armonk, NY, USA). Continuous variables are presented as mean ± standard deviation (SD). Student’s *t*-test was used for comparing pre- and postoperative VAS and OMAS. A two-sided test was deemed statistically significant at *p* < 0.05. Both interobserver and intraobserver reliability were assessed for each evaluation method. The reliability of all methods with continuous variables was assessed using intraclass correlation coefficients (ICCs) calculated with a two-way mixed-effects model for consistency of agreement.

## 3. Results

The study included 21 patients with AITFL avulsion fractures and displaced malleolar fractures. The participants comprised 7 males and 14 females, with an average age of 56.48 years (range: 19–85 years).

### 3.1. Clinical Outcomes

The average OMAS significantly improved from 5.95 preoperatively (range: 0–35 points) to 83.57 postoperatively (range: 60–95 points) (*p* < 0.001). The average VAS score before surgery was 7.95 (range: 7–9 points), and the average VAS score after surgery was 0.19 (range: 0–2 points), also indicating significant improvement (*p* < 0.001). No patient required reoperation because of complications. Table 2 presents an extensive overview of patient demographics and clinical analysis results, detailing the study population’s characteristics and clinical assessment findings.

### 3.2. Radiologic Outcomes

At the final follow-up, simple radiographs confirmed bony union in all cases. Table 3 presents the radiologic measurements obtained by the first and second observers using all evaluation methods, while Table 4 reports the results of agreement analysis. There were no statistically significant differences between the normal side and the injured side in the results of four radiologic measurements taken by the first and second observers (*p* > 0.05). The intraobserver reliability of the four radiological measurements ranged from 0.848 to 0.922 for the first observer and from 0.859 to 0.917 for the second observer. The interobserver reliability of the four radiological measurements across the two observers ranged from 0.936 to 0.963.

### 3.3. Complications

Out of the 21 patients included in the study, only a few experienced complications. Three patients had minor wounds that necessitated further dressing care, while another patient developed a superficial infection that was successfully treated with antibiotics.

## 4. Discussion

The ankle joint complex is made up of the lower leg and the foot, which allows the lower limb to interact with the ground during activities such as walking. The ankle is able to withstand high forces while remaining stable because of its bony and ligamentous structures [22]. These include the anterior talofibular ligament, the posterior talofibular ligament, the calcaneofibular ligament, and the deltoid ligament. These ligaments work together to prevent excessive movement in any direction, helping to maintain proper alignment of the bones within the joint. In addition to its bony and ligamentous structures, the ankle joint complex also includes several muscles that help control movement and provide stability. These muscles include those in the anterior compartment of the leg, such as the tibialis anterior, the extensor hallucis longus, the extensor digitorum longus, and the peroneus tertius; those in the lateral compartment of the leg, such as the peroneus longus and the peroneus brevis; and those in the posterior compartment of the leg, such as the gastrocnemius, the soleus, the plantaris, the popliteus, the flexor hallucis longus, the flexor digitorum longus, and the tibialis posterior. Overall, it is clear that a combination of bony architecture, ligaments, muscles, and other soft tissue structures work together to provide stability to the ankle joint complex. This allows for the smooth and coordinated movement of the lower limb during activities such as walking or running. It is important to maintain proper strength and flexibility in these structures in order to prevent injury and ensure the optimal function of this important joint complex. When the tibia rotates laterally, this movement is transmitted to the talus and then to the fibula. As a result, both the tibia and fibula rotate around their own longitudinal axes. The fibula also rotates around the tibia’s longitudinal axis, guided by the inferior tibiofibular joint. This allows for the smooth and coordinated movement of the ankle joint complex [23]. The stability of the distal tibiofibular syndesmosis is crucial for the proper functioning of the ankle and lower extremities. The ankle’s stability is largely due to the mortise, which is formed by the tibia and fibula around the talus. When the ankle is dorsiflexed, the fibula rotates externally, allowing its wider anterior portion to fill the mortise more completely and maximize contact between the articular surfaces [24]. The distal structures of the lower leg help prevent the lateral displacement of the fibula and talus, ensuring that the mortise remains stable [25]. The junction of the distal tibial ligament comprises the AITFL, the posterior inferior tibiofibular ligament (PITFL), the interosseous ligament, and the inferior transverse ligament. The AITFL, which forms a bridge between the anterior tubercle of the tibia and fibula, is crucial for maintaining the stability of the distal tibial joint, accounting for 35% of its overall stability [5]. The presence or absence of the AITFL significantly influences the treatment strategies and prognosis of ankle-joint injuries [26,27,28].

Ankle fractures are a common injury, accounting for 10.2% of all bone injuries [29]. According to a study by Elsoe R et al. [30], the incidence of ankle fractures was found to be 157.1 annually per 100,000 persons for males and 179.5 annually per 100,000 persons for females. Another study by Juto H et al. [31] found an incidence of 179 adult ankle fractures annually per 100,000 persons. The majority of these fractures were caused by low-energy trauma and were more frequent among females, with an increased incidence mainly between the ages of 30 and 60. Ankle fractures are not typically associated with osteoporosis; earlier studies have concluded that fractures of the wrist, humerus, vertebra, and hip have a significant relationship with low bone mass but ankle fractures do not [32,33]. However, they should still be considered a fragility fracture in the elderly. This is because the incidence of ankle fractures increases markedly with age and is more common among females, as supported by Court-Brown’s classification of ankle fractures as a fragility fracture [29].

Typically, injuries to the distal tibial ligament joint arise from external rotational forces, often coinciding with a supination-external rotation-, pronation-external rotation-, or pronation-abduction-type fracture following the Lauge–Hansen ankle joint classification [34,35,36,37]. The AITFL avulsion fracture, which occurs when the AITFL detaches from the bone, was first chronicled by Wagstaffe in 1875 [38]. Pankovich’s classification (a modification of the Wagstaffe classification) sorts AITFL avulsion fractures into four types: Type I for avulsion fractures on the fibular side; Type II for avulsion fractures accompanied by fibular fractures; Type III for avulsion fractures on the tibial side; and Type IV for bilateral avulsion fractures. Type II fractures are the most common [39,40]. Diagnosing AITFL avulsion fractures using conventional radiography can be challenging given the overlapping tibial structures. However, Park et al. [28] proposed that 45-degree internal rotation radiography could enhance diagnostic accuracy. In our study, we diagnosed the fracture employing AP, lateral, and mortise X-rays of both ankles and 3D CT scans. In a few cases, the diagnosis was established during surgery.

When treating AITFL avulsion fractures, the restoration of the ligament, anatomical reduction, and the robust internal fixation of the ankle joint fracture are essential. Direct fracture fixation not only ensures the bone-to-bone fixation of the anterior syndesmosis but also correctly places the fibula in the tibial incisura. Inaccuracy in AITFL restoration could lead to translational or rotational malposition, which can compromise the ankle mortise’s structure, contribute to chronic pain in the distal tibiofibular joint, induce traumatic arthritis because of chronic talus dislocation, and cause pain resulting from fracture fragment indentation [41,42,43]. While the nonunion of an AITFL avulsion fracture typically resulted in average outcomes, accurately diagnosing and reducing the AITFL avulsion fracture and appropriately treating the accompanying ankle joint fracture produced good results.

Various studies have reported differing success rates for the various treatment options for Chaput fractures. Previous research has explored the outcomes of untreated fractures, the necessity for additional surgery in certain instances, and the results of open reduction/internal fixation and direct avulsion fracture fixation. According to Haraguchi et al. [44], only 65% of non-operated Chaput fractures successfully healed and formed a union. Zhao et al. [45] performed a study on 15 adult patients with ankle fractures, including Tillaux–Chaput fractures. They achieved an 80% success rate with open reduction/internal fixation, and the majority of cases had excellent or good AOFAS scores.

In contrast, Bae et al. [9,10] performed direct avulsion fracture fixation on patients with syndesmotic instability after malleolar fractures and achieved stability in 83.3% of cases, while 16.7% required additional syndesmosis screw fixation. Chung et al. [8] reported positive outcomes using K-wires, mini-screws, or absorbable suture materials, whereas Rammelt et al. [46] employed plates, screws, and suture anchors to achieve the desired results. Gasparova et al. [47] suggested that screw fixation is suitable for single-fragment fractures, while plate fixation is more effective for fractures involving multiple fragments. 

TBW has traditionally been recommended for smaller fractures or cases where screw fixation is not feasible, such as avulsion fractures or in patients with osteoporotic bones [48]. Recent studies have confirmed that larger fragments treated with TBW demonstrate favorable fusion rates and functional outcomes [49]. However, a comprehensive comparison of fixation strength between TBW and other devices is yet to be conducted. This could be a potential avenue for further research, perhaps utilizing cadaver studies.

In conclusion, the direct fixation of Chaput fractures is a viable option that can yield positive results without necessitating extensive surgical expertise.

## 5. Conclusions

This study showcased the outcomes of surgeries employing the TBW technique for treating Chaput fractures. The method provided high fixation strength and enhanced long-term functional outcomes for patients. Although a variety of treatment options are available, TBW is a credible alternative that yields beneficial results for patients with Chaput fractures and associated syndesmosis instability. Further investigations and comparative studies with other fixation devices are necessary to affirm the effectiveness of this technique and refine treatment strategies for these complex ankle fractures.

## Figures and Tables

**Table 1:** Methods used for measurement.

Method	Description
Direct Anterior Difference	This is the perpendicular distance measured from the incisura to the anterior end of the fibular orientation line.
Direct Posterior Difference	This refers to the perpendicular distance from the incisura to the posterior end of the line representing the fibular orientation.
Fibular Translation	This is the distance measured between the anterior border of the tibial incisura and a line representing the direct anterior difference. It is considered positive when the fibula is situated behind the anterior border of the incisura.
Fibular Rotation	This is the angle formed between a line connecting the anterior and posterior borders of the tibial incisura and another line on the fibula indicating its orientation. This angle is deemed positive when the fibula is internally rotated relative to the incisura.

**Table 2:** Patient demographics and results.

Pt. No.	Age	Sex	Cause	Lauge-Hansen Classification	Injured Side	OMAS		VAS Score	
						Pre	Post	Pre	Post
1	57	F	S	SER IV	Left	30	80	8	1
2	76	F	S	SER IV	Left	25	85	7	0
3	56	F	S	SER II	Right	30	90	8	0
4	39	M	S	SER IV	Left	35	95	8	0
5	58	F	S	SER IV	Left	0	80	9	1
6	79	F	TA	PER IV	Left	0	70	8	0
7	53	F	S	SER IV	Left	0	60	9	0
8	67	M	TA	PER IV	Right	0	95	8	0
9	19	M	TA	PER II	Right	0	90	7	0
10	47	F	S	SER II	Left	0	80	8	0
11	57	M	S	PER III	Left	0	90	8	0
12	68	F	S	SER IV	Left	0	75	7	0
13	61	M	TA	SER IV	Right	0	90	7	0
14	62	F	S	SER II	Left	0	85	8	1
15	31	F	S	SER III	Right	0	85	8	0
16	85	M	TA	SER IV	Right	0	80	9	0
17	64	F	S	SER II	Left	5	85	8	0
18	54	F	S	PER IV	Right	0	85	7	1
19	55	F	S	SER II	Right	0	90	8	0
20	42	M	S	SER IV	Left	0	85	9	0
21	56	F	S	SER IV	Left	0	80	8	0
Mean	56.48	NA	NA	NA	NA	5.95	83.57	7.95	0.19
SD	15.39	NA	NA	NA	NA	12.11	8.24	0.67	0.40
*p* value							<0.001		<0.001

Abbreviation: Pt. No., patient number; OMAS, Olerud–Molander Ankle Score; VAS, visual analog scale; Pre, preoperative; Post, postoperative; F, female; M, male; S, slip down; TA, traffic accident; SER, supination external rotation; PER, pronation external rotation NA, not applicable; SD, standard deviation.

**Table 3:** Results of four evaluation methods for the first and second observers.

Evaluation Method	Observer 1			Observer 2		
	Normal Side (*n* = 21)	Injured Side (*n* = 21)	*p*-Value ^2^	Normal Side (*n* = 21)	Injured Side (*n* = 21)	*p*-Value ^2^
Direct anterior difference, mm	4.28 (0.84) ^1^	4.84 (1.36)	0.114	4.29 (0.87)	4.93 (1.38)	0.082
Direct posterior difference, mm	7.70 (0.85)	7.78 (1.31)	0.830	7.71 (0.83)	7.78 (1.28)	0.831
Fibular translation, mm	1.47 (0.52)	1.61 (0.53)	0.380	1.51 (0.51)	1.64 (0.57)	0.430
Fibular rotation, deg	8.56 (3.96)	8.48 (4.13)	0.948	8.62 (3.90)	8.47 (4.06)	0.902

^1^ Values are mean (SD). ^2^ There were no statistically significant differences between the normal side and the injured side in the results of four radiologic measurements taken by the first and second observers (*p* > 0.05).

**Table 4:** Assessment of intraobserver and interobserver reliability of evaluation methods between the first and second observers.

Evaluation Method	Intraobserver Reliability		Interobserver Reliability
	Observer 1	Observer 2	
Direct anterior difference, mm	0.885	0.886	0.950
Direct posterior difference, mm	0.848	0.859	0.936
Fibular translation, mm	0.922	0.917	0.963
Fibular rotation, deg	0.902	0.900	0.957

## Data Availability

Data sharing is not applicable to this article as no datasets were generated or analyzed during the current study.
